# Multiple Sclerosis Treatment and Melanoma Development

**DOI:** 10.3390/ijms21082950

**Published:** 2020-04-22

**Authors:** Maria Luigia Carbone, Pedro Miguel Lacal, Serena Messinese, Laura De Giglio, Carlo Pozzilli, Severino Persechino, Cinzia Mazzanti, Cristina Maria Failla, Gianluca Pagnanelli

**Affiliations:** 1 Laboratory of Experimental Immunology, IDI-IRCCS, 00167 Rome, Italy; marialuigia.carbone@idi.it; 2 Laboratory of Molecular Oncology, IDI-IRCCS, 00167 Rome, Italy; p.lacal@idi.it; 3 I Dermatology Department, IDI-IRCCS, 00167 Rome, Italy; s.messinese2@idi.it (S.M.); c.mazzanti@idi.it (C.M.); g.pagnanelli@idi.it (G.P.); 4 Medicine Department, Neurology Unit, San Filippo Neri Hospital, 00135 Rome, Italy; lauradegiglio@gmail.com; 5 Department of Human Neurosciences, Sant’Andrea Hospital, MS Centre, Sapienza University, 00189 Rome, Italy; carlo.pozzilli@uniroma1.it; 6 NESMOS Department, Dermatology Unit, Sant’Andrea Hospital, Sapienza University, 00189 Rome, Italy; severino.persechino@uniroma1.it

**Keywords:** melanoma, fingolimod, natalizumab, VEGF-A

## Abstract

Therapy of multiple sclerosis (MS) with disease-modifying agents such as natalizumab or fingolimod has been associated with the development of cutaneous melanoma. Here we briefly revise literature data and report of a case of a 48-year old woman who developed a melanoma and several atypical naevi after sub sequential treatment with natalizumab (1 year) and fingolimod (7 years). By immunohistochemistry we observed the presence of T cells and leukocyte infiltration as well as of vascular endothelial growth factor (VEGF)-A expression in the patient melanoma biopsy. Then, we analyzed proliferation, migration and VEGF-A expression in three melanoma cell lines and found out that both natalizumab and fingolimod inhibited tumor cell proliferation but promoted or blocked cell migration depending on the cell line examined. VEGF-A secretion was augmented in one melanoma cell line only after fingolimod treatment. In conclusion, our in vitro data do not support the hypothesis of a direct action of natalizumab or fingolimod on melanoma progression but acting on the tumor microenvironment these treatments could indirectly favor melanoma evolution.

## 1. Introduction

Recent innovations in disease treatment markedly improved expectancy for multiple sclerosis (MS) patients, reducing their disability burden and improving the quality of life. In fact, several drugs are now available able to modify the pathology course, limiting both relapses and disease progression [1]. However, a careful balance between benefits and risks must be done on individual basis since the more effective treatments often cause themselves severe adverse effects.

Natalizumab (Tysabri) and fingolimod (FTY720, Gilenya), are among the most effective and diffuse therapies for patients with MS. Natalizumab is a humanized monoclonal antibody directed against the α4 integrin subunit that is expressed on T and B lymphocytes, monocytes, macrophages, natural killer (NK) and dendritic cells. By blocking α4 integrin, natalizumab interferes with immune cell migration across the blood brain barrier inhibiting trans-endothelial migration to the central nervous system [2]. Natalizumab shows important anti-inflammatory responses and neuroprotective effects, but it enhances the risk of developing a rare brain infection, the progressive multifocal leukoencephalopathy. Other side effects include hepatotoxicity, allergic reactions and a higher risk of infection [2].

Fingolimod is a non-specific small molecule acting as a sphingosine-1-phosphate receptor modulator, causing receptor internalization and leading to a redistribution of circulating lymphocytes into secondary lymphoid organs thereby inducing a state of peripheral lymphopenia. Thus, fingolimod reduces infiltration of autoreactive lymphocytes into the central nervous system [3]. The sphingosine-1-phosphate receptor is a potent inducer of endothelial cell chemotaxis and phosphorylated fingolimod, acting as a receptor agonist, induces endothelial cell migration, adherens junction assembly and inhibits vascular endothelial growth factor (VEGF)-A-mediated vascular permeability [4]. Fingolimod-mediated loss of sphingosine-1-phosphate receptor from the astrocytes attenuates neuroinflammation, demyelination and axonal damage [5]. However, fingolimod treatment in MS has been linked to herpetic infections, cardiac and hepatic adverse effects [3].

There are reports in the literature about the development of cutaneous melanoma following MS therapy with either natalizumab [6,7,8,9,10,11,12,13] or fingolimod [14,15,16,17,18,19] but the number of cases was low and did not reach any statistical significance. Therefore, melanoma occurrence in these patients could be merely a coincidence [20]. Nevertheless, recent studies indicated a statistically significant association of melanoma occurrence with the treatment with those disease modifying drugs [21,22]. Prospective follow-up of MS patients treated with natalizumab evaluated possible modifications of naevi under treatment and found out that either after 14 months [23] or after 4 years [24] the degree of clinical and dermoscopic changes during natalizumab therapy did not differ from the rate of spontaneous evolution of naevi in untreated individuals as reported in the literature. These data suggest that inhibition of α4β1 integrin does not directly promote malignant transformation of melanocytes.

Fingolimod behaves as an antiangiogenic drug [25] and inhibits tumor growth and metastatic spreading in vivo in murine melanoma and breast cancer models [25,26]. Moreover, fingolimod induces apoptosis in vitro in mouse melanoma cells and blocks metastasis spreading both in a syngeneic mouse model [27] or in canine melanoma [28]. However, a protumorigenic role of fingolimod was also proposed, with this drug acting by enhancing accumulation of myeloid-derived suppressor cells (MDSCs) around the tumor lesion [29].

Natalizumab could affect melanoma progression through different mechanisms. When the α4 integrin subunit was introduced into a murine melanoma cell line, its expression significantly reduced the melanoma invasive potential both in vitro in a Matrigel assay and in vivo in a melanoma mouse model [30]. Thus, natalizumab-mediated blockage of the α4 integrin could prompt the invasive stage of metastasis formation. Conversely, other reports indicated that α4β1 integrin is frequently over-expressed in highly invasive melanoma cells and integrin inhibition could prevent metastasis spreading [31]. In addition, in vitro prolonged treatment of human NK cells, which express the α4β1 integrin, resulted into impaired NK cell degranulation towards melanoma cells. Similarly, NK cell migration in the direction of melanoma cells was significantly reduced in the presence of natalizumab. Finally, α4β1 integrin expression was diminished by natalizumab treatment in vitro as well as in MS patients, decreasing with time of natalizumab therapy, suggesting that this drug could alter NK-mediated immune surveillance against melanoma with a protumorigenic outcome [32].

## 2. Results

### 2.1. Patient Clinical Findings

A 48-year old woman affected by MS from the age of 28 received treatment with natalizumab for 1 year and then, due to the development of anti-natalizumab antibodies, with fingolimod. During the following seven year-long treatment with fingolimod, the patient developed a thin cutaneous melanoma (pT1a, 0.2 mm) and several atypical naevi. The patient presented herself with numerous naevi throughout the body and reported of a familiarity with epithelial tumors and autoimmune diseases as her grandfather developed a prostate tumor and amyotrophic lateral sclerosis. The patient reported also sun burning when she was a child.

As indicated in Table 1, cases of cutaneous, mucosal or ocular melanoma reported after MS therapy with natalizumab or fingolimod often presented with concomitant well-known risk factors for melanoma development. In addition to our patient, three other patients were reported in a Dutch study who underwent to subsequent MS treatment with natalizumab first and then fingolimod and developed superficial spreading melanoma after at least one-year therapy with fingolimod [18].

**Figure 1:**
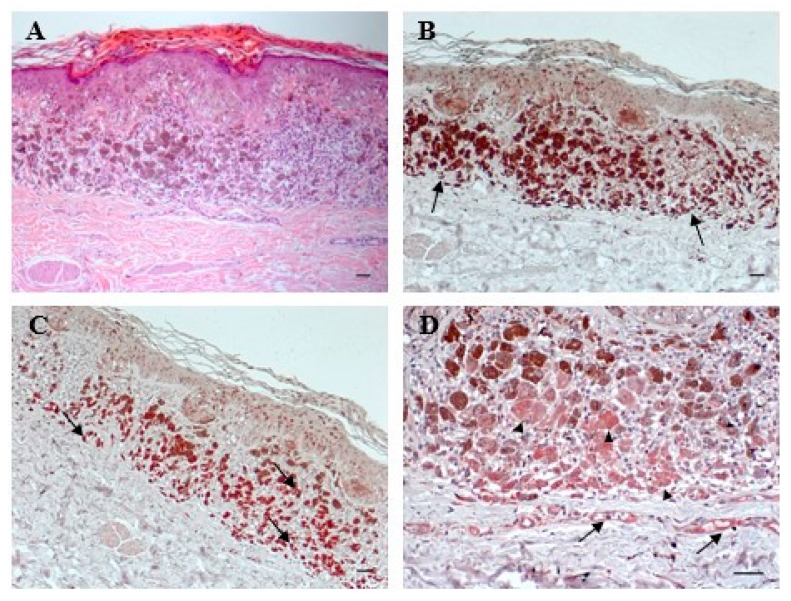
Immunohistochemical analysis of patient melanoma biopsy. (**A**) Hematoxylin–eosin staining of the lesion. Immunohistochemical staining of the melanoma lesion using an anti-CD3 antibody (**B**), an anti-CD11b antibody (**C**) or an anti-VEGF-A antibody (**D**). Bars = 200 µm. Arrows indicate positive staining of infiltrating cells in (**B**,**C**) or of endothelial cells in (**D**). Arrowheads indicate VEGF-A staining in the melanoma cells in (**D**).

Hematoxylin–eosin staining of the patient primary melanoma biopsy is shown in Figure 1, panel A. Then, we analyzed the same specimen by immunohistochemistry. Around the melanoma lesion a CD3 positive infiltrate of T lymphocytes was observed (Figure 1B) as well as a CD11b positive infiltrate that identified the presence of monocytes, macrophages and granulocytes or MDSCs (Figure 1C).

Expression of VEGF-A was also present in the melanoma cells and in the endothelial cells of the dermal vessels surrounding the lesion (Figure 1D).

### 2.2. Melanoma Cell Treatment with Fingolimod or Natalizumab

**Figure 2:**
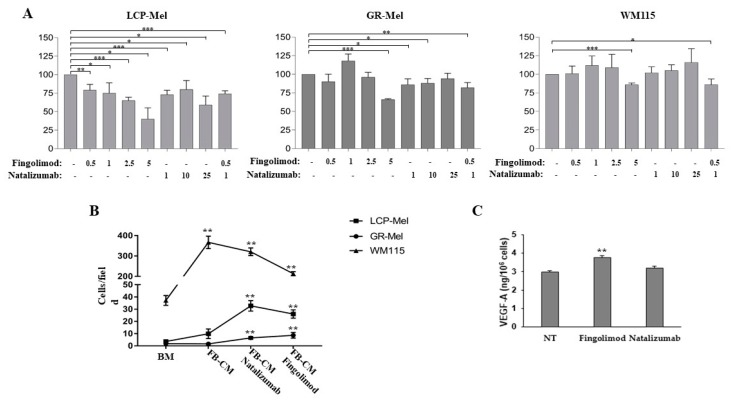
Fingolimod and natalizumab effects on human melanoma cells. (**A**) Proliferation assay of the three melanoma cell lines after a 24-h treatment with the indicated concentration of fingolimod or natalizumab. Results are reported as the percent of differences ± standard deviation (SD) in the ATP levels as compared to the not treated control considered as 100%. * *p* ≤ 0.05; ** *p* ≤ 0.01; *** *p* ≤ 0.005 referred to not treated cells. (**B**) Cell migration of the three melanoma cell lines towards basal medium (BM), or fibroblast-conditioned medium (FB-CM) as a nonspecific stimulus, in the absence or in the presence of natalizumab or fingolimod. Results are expressed as the mean number of migrated cells ± SD. ** *p* ≤ 0.01 referred to BM. (**C**) ELISA for VEGF-A secretion in the supernatant of LCP-Mel cells not treated (NT) or treated with fingolimod (5 µM) or natalizumab (20 µg/mL). Results are reported as ng/10^6^ cells ± SD. ** *p* ≤ 0.01 referred to not treated cells (NT).

To understand whether fingolimod and/or natalizumab therapy could influence melanoma progression, we studied in vitro three different human melanoma cell lines (LCP-Mel, GR-Mel and WM115) derived from primary tumors. We performed a proliferation assay using diverse concentrations of the two drugs for 24 h. As reported in Figure 2, fingolimod treatment, starting from a concentration of 0.5 µM, significantly impaired LCP-Mel proliferation whereas it did not affected proliferation of both GR-Mel and WM115 cells until a concentration of 5 µM fingolimod is reached (Figure 2A). Natalizumab treatment impaired proliferation of LCP-Mel and GR-Mel starting from a concentration of 1 µM, whereas it did not affect WM115 cell proliferation. Interestingly, combined treatment with 0.5 µM fingolimod plus 1 µM natalizumab partly affected also WM115 cell proliferation (Figure 2A).

Then, we investigated whether fingolimod or natalizumab could affect human melanoma cell migration. The three cell lines behaved differently, probably due to a diverse response to stimulation with FB-CM. In the case of GR-Mel and LCP-Mel, which showed a reduced ability to migrate in response to the nonspecific stimulus, cell migration significantly augmented after treatment with either drug, fingolimod or natalizumab. On the other hand, the highly responsive WM115 cell line showed a lower migration rate, in respect to stimulation with FB-CM alone, upon treatment with natalizumab and even lower when treated with fingolimod (Figure 2B).

Considering the presence of VEGF-A in patient melanoma specimen that we observed by immunohistochemistry, we also analyzed VEGF-A secretion in these melanoma cell lines after treatment with fingolimod or natalizumab. As reported in Figure 2C, treatment with fingolimod, but not with natalizumab, significantly augmented VEGF-A secretion in LCP-Mel melanoma cells. Both GR-Mel and WM115 secreted undetectable amount of VEGF-A in basal condition, as previously reported [33], and treatment with these drugs did not increase the angiogenic factor secretion.

## 3. Discussion

The debate on a possible relationship between cutaneous melanoma and MS therapy with natalizumab and/or fingolimod is still open. Since the first two reported cases in 2008 [9], patient reports [6,8,11,12,13,14,15,16,17,18] and evaluation of clinical trial results [19,21,22] indicate that an association between melanoma development and treatment with these drugs may occur, but the number of cases examined is too low to exclude a mere casualty. In vitro analysis using cell lines and preclinical melanoma animal models did not help in clarifying this aspect since also in these conditions natalizumab or fingolimod act either as protumorigenic or as antitumorigenic molecules. Therefore, a conclusion on the relationship between these drugs and melanoma has not been drawn yet.

Here we reported a case of a woman affected by MS who developed a cutaneous melanoma and different atypical naevi after 1-year treatment with natalizumab followed by 7-year therapy with fingolimod. She presented herself with multiple naevi and a sunburn in childhood. Therefore, as reported for other cases [7,8,9,12,18,19] and underlined here, she had well-known risk factors for melanoma development, suggesting that occurrence of melanoma was not related to the therapy.

We also performed the first immunohistochemical analyses of such a melanoma lesion and found out the presence of a leucocyte infiltrate around the tumor. This finding was unexpected since the patient was treated with immunomodulating drugs. However, the subtype of leukocyte infiltration was not examined and the presence of immunosuppressing cells such as regulatory T-lymphocytes or MDSCs is probable. An immunosuppressive environment could also result from VEGF-A expression [34]. The presence of a positive staining for members of the VEGF family of growth factors is rarely seen in thin cutaneous melanomas, since their expression often increases with tumor thickness as the result of an “angiogenic switch” in the tumor cells towards a more aggressive phenotype [33]. Altogether, these conditions indicate that our patient developed a high-risk melanoma with an unfavorable prognosis.

Then we performed some experiments on three different melanoma cell lines derived from primary tumors. It is of note that most of the previously reported studies on natalizumab or the fingolimod role in melanoma were performed in vitro on the murine B16F10 cell line or on a single human cell line. We found that both fingolimod and natalizumab inhibit melanoma cell proliferation in a dose-dependent way. These data agree with what has been previously observed in other tumor cells [25,26,27,28]. However, we noted that each melanoma cell line behaved differently in response to treatment with increasing drug doses. Correspondingly, we found that natalizumab or fingolimod treatment could either inhibit or stimulate melanoma cell migration depending on the cell line analyzed. Therefore, our data indicate only a tendency of these drugs in inhibiting cell proliferation and migration but could not demonstrate a general and unique response of melanoma cells to treatment. Our data also underline that it is extremely important to test drug effects on more than one tumor cell line, otherwise the great diversity of human melanoma cells would not permit to consider the data as conclusive.

Finally, we found for the first time that secretion of VEGF-A by melanoma cells was influenced by fingolimod, while it was not dependent on natalizumab treatment. Fingolimod was previously considered as an antiangiogenic compound able to block both VEGF-A- and sphingosine-1-phosphate receptor-mediated angiogenesis and vessel permeability [4,25]. However, induction of VEGF-A secretion by fingolimod in melanoma cells supports both our in vivo findings and a possible role of fingolimod in melanoma “angiogenic switch”.

Taken together, our in vitro data indicate that in general neither fingolimod nor natalizumab directly act on melanoma cells to prompt proliferation or invasion but have instead an antitumorigenic action. A possible indirect action could be rather executed by fingolimod through induction of VEGF-A expression and recruitment of immune-suppressive MDSCs [29] and by natalizumab through impairment of NK cell functions [32]. Therefore, treatment with these drugs can modify tumor microenvironment towards an immune-suppressive, proangiogenic one, favoring melanoma progression. It will be highly informative in the future to analyze this specific aspect both in atypical naevi and in tumor specimens of melanomas that would occur in fingolimod- or natalizumab-treated MS patients.

## 4. Materials and Methods

### 4.1. Patient’s Samples and Immunohistochemistry

Patient’s melanoma biopsy was collected in the IDI-IRCCS histopathology archive after patient signature of an informed consensus. The study was approved by IDI-IRCCS Ethic Committee (497-2, 10 July 2017). Five micrometer sections were dewaxed and rehydrated. Hematoxylin–eosin staining was performed using a standard protocol. For immunohistochemistry, endogenous peroxidase was quenched, and antigen retrieval was performed by heat exposure. After incubation with a blocking buffer (Dako, Santa Clara, CA, USA) for 1 h, sections were treated with the anti-human VEGF-A mouse monoclonal antibody (Beckton Dickinson), at a concentration of 5 µg/mL; with the anti-human CD3 rabbit antibody (A0452 Dako), diluted 1:100, or with the anti-human CD11b rabbit monoclonal antibody (EPR1344, Abcam, Cambridge, UK), diluted 1:100. Secondary biotinylated monoclonal Abs and staining kits were obtained from Vector Laboratories (Burlingame, CA, USA). Immunoreactivity was visualized with peroxidase reaction using 3-amino-9-ethylcarbazole (AEC, Vector Laboratories) and sections were counterstained with hematoxylin. As negative controls, primary Abs were omitted. Stained sections were analyzed with AxioCam digital camera attached to an Axioplan 2 microscope (Carl Zeiss AG, Oberkochen, Germany).

### 4.2. Melanoma Cells

Of the three human melanoma cell lines used, GR-Mel was established in our laboratory, WM115 was purchased from the American Type Culture Collection (ATCC, Rockville, MD, USA), and LCP-Mel was a gift from Dr. F. Guadagni (Istituto Regina Elena, Rome, Italy). Melanoma cells were grown in RPMI 1640 medium supplemented with 10% fetal bovine serum, 2 mM glutamine, and 50 mg/mL gentamycin (Euroclone, Oud-Beijerland, Holland).

### 4.3. Proliferation and Migration Assays

Melanoma cell proliferation in the presence or absence of the indicated concentration of fingolimod (FTY720, Sigma Aldrich, Saint Louis, MO, USA) or natalizumab (TYSABRI, Biogen Manufacturing ApS, Denmark) was evaluated after 24 h in 96-well plates using the ATPlite Assay from PerkinElmer (Waltham, MA, USA), following the manufacturer’s instructions.

Melanoma cells were incubated for 24 h with fingolimod (5 µM) or natalizumab (20 µg/mL). Cells were detached from the plates with phosphate-buffered saline (PBS)/ethylenediamine tetraacetic acid (EDTA) and an equal number of viable cells was loaded on Boyden chambers equipped with 8 µm pore diameter polycarbonate filters (Nuclepore, Whatman Incorporated, Clifton, NJ, USA) coated with 5 µg/mL gelatin. The assay was carried on for 18 h as previously described [35]. Migrated cells were counted under the microscope (200× magnification). Human fibroblast conditioned medium (FB-CM), used as a non-specific stimulus for the cell migration assay, was obtained by incubating semi-confluent cell cultures in 0.1% BSA/RPMI-1640 medium for 24 h. Supernatant was then collected, aliquoted and frozen until use.

### 4.4. VEGF-A ELISA

Melanoma cells were grown in complete medium until semiconfluency was reached. Then, cultures were incubated for 24 h in RPMI medium without fetal bovine serum and with the addition of 0.1% BSA. After cell treatment with fingolimod (5 µM) or natalizumab (20 µg/mL) for additional 24 h, supernatants were collected and concentrated at least 10-fold in Centriplus concentrators (Amicon, Beverly, MA, USA). Cells were detached with PBS/EDTA and counted to normalize VEGF-A secretion by the total number of cells. ELISA was performed using the goat anti-VEGF-A antibody (R&D Systems, Minneapolis, MN, USA) at a 10 µg/mL concentration in PBS to coat Maxisorp Nunc immunoplates (Nunc, Roskilde, Denmark). Detection was performed with the biotinylated goat anti-VEGF-A antibody (R&D Systems) and streptavidin alkaline phosphatase conjugate (1:10,000) (Boehringer Ingelheim, Ingelheim am Rhein, Germany). Optical density was then measured at 405 nm in a Microplate reader 3550-UV (Bio-Rad, Hercules, CA, USA).

### 4.5. Statistical Analyses

Statistical analysis was performed using Wilcoxon’s signed rank test (GraphPad Prism v.7.03 Software, La Jolla, CA, USA) for proliferation and migration assays and a paired two-tailed Student’s *t*-test for ELISA. Values of *p* ≤ 0.05 were considered as statistically significant.

## Figures and Tables

**Table 1:** Reported cases of melanoma after multiple sclerosis (MS) therapy.

Case Numbers	Therapeutic Treatment	Risk Factors
1	Natalizumab [6]	NR
1	Natalizumab [7]	Pre-existing lesion
1	Natalizumab [8]	Many atypical naevi
2	Natalizumab [9]	Many atypical naevi and familiarity
1	Natalizumab [10]	NR
1	Natalizumab [11]	NR
1	Natalizumab [12]	Many atypical naevi
1+137	Natalizumab [13]	NR
3	Fingolimod [14]	NR
1	Fingolimod [15]	NR
1	Fingolimod [16]	None
1	Fingolimod [17]	NR
5	Fingolimod [18]	Fair skin and several large naevi (3 out of 5 patients)
1	Fingolimod [19]	Many naevi

NR = not reported.

## Abbreviations

MS	Multiple sclerosis
VEGF	Vascular endothelial growth factor
NK	Natural killer
MDSCs	Myeloid-derived suppressor cells
